# Books and Authors

**Published:** 1912-02

**Authors:** 


### BOOKS AND AUTHORS

**Diseases of the Ear, Nose and Throat,** Medical and Surgical, **Dr. Wendell Christopher Phillips,** New York; published by the F. A. Davis Co., Philadelphia, 1911. 847 pages, 545 illustrations, 31 full page plates. $6.00.

One hundred pages and 100 illustrations are devoted to the diseases of the mastoid process. This is a good index of the thoroughness of the work. The author has the knack of getting at the root of things and his book is large, not because of prolixity but of abundance of real material.

**Scientific Features of Modern Medicine, Frederic S. Lee,** Ph.D., Dalton Professor of Physiology, Columbia University. Columbia University Press, 1911. 12mo, cloth, 183 pages, $1.50.

This book is a collection of one series of Jesup lectures. Such work as this, presented by a man in close touch with scientific medical progress but not himself a member of the medical profession, is of the utmost value in two ways: To the laity, it gives a fair and much needed appreciation of what modern medicine stands for; to the practitioner, it affords a broad view of fundamental principles and actual accomplishments, all the more valuable because not obscured by the detailed superstructure of medicine as an art.

---

**Minor Surgery, Leonard A. Bidwell,** F.R.C.S., London; published by the University of London Press, 1911. 265 pages, 88 illustrations $2.00.

This work includes repair of tendons, technic of injections as of "606," tuberculin, vaccinations, fractures and many details not formerly considered *minor*. While books of this sort suggest to the sarcastic reviewer, the fable of the ancient philosopher who cut a doorway for his cat and a small one for the kitten, there is no question but that they fulfill an actual demand and perform a useful service, especially when so well composed as the present.

---

**Anaesthesia and Analgesia, J. D. Mortimer,** M.B., M.R.C.S., London; published by the University of London Press, 1911, 276 pages, 29 illustrations, $2.00.

This book is conspicuous by its omission of many complicatea modern apparatus for the administration of new anaesthetics. It adheres to conservative views, as the general safety of aether; the limitations of spinal anaesthesia. It omits, also, many recent local analgesics. But, it shows thorough experience with the subject, sound judgement and careful attention to detail. The reader may, at first be disappointed with the omissions indicated but he will be impressed that this author, who is not too hurried to spell anaesthesia with a diphthong and who writes his name with initials instead of after the modern fashion, would be a safe man to administer an anaesthetic and that he is a safe teacher to follow. We have old-fashioned prejudices on this subject, strengthened by the death from carbon dioxid, of a patient operated on under the most modern circumstances. The apparatus was all right but the assistant got a trifle confused with the valves and the operator was one of the kind who did not want germ-laden internists and family doctors distracting his attention by fussing about the heart and respiration, liability to chill from exposed wet surfaces, etc.

**The Blood and Its Third Anatomical Element.** Application of the Microzymian Theory of the living organization to the study of the anatomical and chemical constitution of the blood and to that of the anatomical and physiological causes of the phenomena of its coagulation and of its other spontaneous changes. By **A. Bechamp,** formerly Professor in the Medical Faculty of Montpelier (France) corresponding member of the Academy of Medicine, etc. Translated from the French by **Montague R. Leverson,** M.D., of the Baltimore Medical School and M.A. and Ph.D. of the University of Gottingen. 438 pages. Cloth, $1.50. Postage, 10 cents. Philadelphia, Pa. Boericke & Tafel. 1911.

Two of the staff of the BUFFALO MEDICAL JOURNAL have vainly tried to review this book. Its venerable author, who died in 1908, aged nearly 92, holds such different conceptions of histology and pathology from those which we have been taught, that his work is incomprehensible. We say this in no disrespectful spirit and with no arrogance. One of our homoepathic exchanges dodges the issue in much the same way, though more critically. There is much that is of great interest in the book aside from the fact that a general upsetting of preconceived notions is wholesome mental gymnastics. The results of a life time of study by a scholarly man, along lines of thought widely diverging from those of the majority of pathologists, deserve, we think, the serious attention of experts. There is always the possibility that the majority view is not the right one and, if it is, the trained student should get many hints for research from one who disagrees with the majority.

---

**Case Histories in Medicine,** illustrating the diagnosis, prognosis and treatment of disease, by **Richard C. Cabot,** M.D., Boston. Published by **W. M. Leonard,** Boston, 1911. Second edition, 295 pages, cloth, $3.00.

We have already reviewed several books of the Case History Series and can only reiterate the favorable opinion regarding the clinical practical method of thus presenting problems in medicine in orderly sequence. This method not only teaches facts, it teaches the reader to think. Dr. Cabot explores and maps out unfamiliar fields of medicine and discovers new and valuable facts in fields already well known by name, in the same way that his ancestors added to the world's knowledge of geography and made possible various economic developments.

---

**Further Researches into Induced Cell-Reproduction and Cancer, H. C. Ross,** M.R.C.S., L.R.C.P., London, **E. H. Ross** (idem), and **J. W. Cropper,** M.B., M.Sc., Liverpool. The McFadden Researches, published by **John Murray,** London; represented by P. Blakiston's Son and Co., Philadelphia. 63 pages, illustrated, $1.00.

Not only is there much difference of opinion regarding cell reproduction and allied experimental findings but there has developed considerable acrimony regarding the relations of the authors to a committee as to whose exact constitution and authority, we are not definitely informed. At any rate, the investigations are of great interest and the contribution is of especial value while the nature of cancer is being threshed out.

---

**Byron Robinson,** A Memorial, including the addresses and tributes presented at a public meeting at the Whitney Opera House, May 22, 1910.

Byron Robinson's contributions to gynaecology and to the study of the sympathetic system are well known and highly appreciated. Few of us who have read his works, in whole or part, have been able to do them full justice or to do ourselves full justice in their application because of lack of knowledge of the subject. He was a tireless worker, closely approaching the German type in his diligence and attention to detail. The one thing for which he lacked time, was to put forth his results in such a way as to appeal to the average, lazy or otherwise engaged American student who likes to have his mental pabulum predigested. For these reasons, we believe that the true appreciation and utilization of his works remains for the future.

---

**A Text Book of Alkaloidal Therapeutics,** by **W. F. Waugh,** M.D., and **W. C. Abbott,** M.D., third edition, published by the Abbott Press, Chicago, 1911. 762 pages.

The key note of so-called alkaloidal therapeutics, a somewhat inclusive term, is to use "the smallest possible quantity of the best obtainable means to produce a desired therapeutic result." "Alkaloidal," therefore, refers not only to alkaloids in the strict sense, but to glucosides, to concentrated extracts when single active principles are not available and to many definite chemic substances now derived from crude plants and animal organs. The book, therefore, may be described as a work on materia medica and therapeutics with crude drugs and Galenicals omitted. With a very brief introduction, the work is arranged alphabetically, ending with a reference table arranged by disease names and referring to drugs of use in each condition, and with a long list of authorities quoted.

It is, in a sense, unfortunate, that the movement toward the use of pure, concentrated drugs, has depended largely on commercial interests; still more unfortunate that this fact has in some quarters aroused a prejudice against such therapeutics and an unwillingness to give credit for the scientific and practical value of the work accomplished for the profession itself. The fact that the business has prospered should not blind us to the fact that both the medical profession and the laity have benefited by the availability of active drugs, in such form as minimize the negative danger of deterioration and the positive danger of overdose. Whle these dangers become progressively less as pharmaceutic and medical education becomes more thorough, there is always danger from a powerful drug in bulk, either through mislabeling, failure to read a label or confusion as to the proper dosage. With drugs in convenient units, each representing an average single dose, these dangers are reduced to a minimum.

In addition, it may be said that the book contains, in convenient form, either for study or reference, a vast amount of therapeutic information, sufficient indeed for all but the occasional moot point.

**Diseases of Children for Nurses.** Including Infant Feeding, Therapeutic Measures Employed in Childhood, Treatment for Emergencies, Prophylaxis, Hygiene and Nursing. By **Robert S. McCombs,** M.D., Assistant Physician to the Dispensary and Instructor of Nurses at the Children's Hospital of Philadelphia. Second edition revised. Octavo of 470 pages, illustrated. Philadelphia and London: W. B. Saunders Company, 1911. Cloth, $2.00 net.

The book had its foundation in the lectures given nurses at the Children's Hospital in Philadelphia, and has been amplified to suit the needs of the nurse anywhere. It has a short description of each disease found in early childhood, with enough anatomy and pathology to give an idea of the body structure and the changes caused by disease. Some treatment is given where it seemed that intelligent application of the remedial measure justified it. Treatment of emergencies is much fuller. Prophylaxis, infant feeding, and infant nursing are given full and detailed space. Intestinal diseases and artificial feeding have full space. Nurses trained in hospitals which do not devote special attention to children are weak on these points, and we know that this book will set them right on many little but vitally important points by which they will often be judged by both the laity and by the physician.

---

**The Anatomic Histological Process of Bright's Disease, and Their Relation to the Functional Changes.** Lectures delivered in the Russell Sage Institute of Pathology, City Hospital, New York, during the winter of 1909, by **Horst Oertel,** Director of the Russell Sage Institute of Pathology. Illustrated. Published by W. B. Saunders Company, Philadelphia and London. Price, $5.

Contains 45 figures and plates. The text is a veritable post-graduate course in the histology and pathology of Bright's disease. As a whole, it is a deftly interwoven word picture of conditions and relations which are inseparable in practice, but which are widely separated in the ordinary method of studying pathology and histology. This scheme is unique, not being attempted in any other work printed in English, and is very effective in impressing the salient points upon the mind of the reader. There are five lectures; the first deals with Historical Introduction and Classification. The second considers The Structure of the Normal Kidney and the Different Views on Its Functions in their Relation to the Pathological Variations. The third takes up The Degenerative and Exudative Features of Nephritis. The fourth, The Results and Terminations of Degenerative and Exudative Nephritis; Productive Changes in the Kidney. The fifth, Productive Nephritis; Changes in Other Viscera; Edema. There is a complete index of names, and also one of subjects. It has an appendix, giving classification of Nephritis and the non-inflammatory lesions of the kidney, wrongly grouped as nephritis. A full list of notes and references is given. Every doctor who feels that there is anything about the histologic pathology of Bright's Disease that he does not know, should read this book; it is a gem suited to any graduate in medicine who seeks to improve his knowledge.

---

**The Diagnostics of Internal Medicine** by **Glentworth Reeve Butler,** M.D., Sc.D., LL.D., Brooklyn; published by D. Appleton & Co., New York, 1911, 3d revised edition, 1193 pages, 5 colored plates, 272 illustrations, **$**

It must cause some reviewers a feeling of shame to remember that the first edition of this book was condemned as indecent because, in preparing charts of the superficies of the body, the author employed professional models in a business like way and had the illustrations reproduced by modern methods. Naturally, as posing for medical purposes is a very minor part of a model's business, the models were attractive and the reproduction introduced, as it were, a personal element not present in a rough sketch or in a painting. Fortunately, this stage of prudery has passed.

We like to watch the growth of a work through successive editions, especially a work like this or Simon's, which struck out into comparatively untried fields and which represents so large a factor of personal hard work. While the present book contains comparatively little of personal invention or discovery, the tabulations of scattered contributions, the bringing into logical order of unsystematic observations, the recognition of relations between apparently unrelated phenomena, warrants us in characterizing the work as original. From the reader's standpoint, the work may be regarded as complete, covering, indeed, a far wider range of disease than would be expected and entering into each diagnostic discussion with a surprising fullness. But, from personal experience, we hazzard the guess that the author's copy is already interlineated and contains inserts for a still more complete future edition.

**Orthopedic Surgery, Dr. E. H. Bradford,** Surgeon to the Boston Children's Hospital, etc., and **Dr. R. W. Lovett,** Associate Surgeon to the Boston Children's Hospital, etc. Publishers, William Wood & Co., 1911. 410 pages; many original illustrations. $3.50.

In this new edition of their well known textbook, the authors have condensed and eliminated until the book is truly a handbook of ready reference, in which students and practitioners can find brief statements of the generally accepted opinions as to the nature and treatment of orthopedic affections. No theories or discussions are introduced and in treatment there are given chiefly such measures as have been proved of value to the workers in Boston. References are made to many articles or books where the reader may find fuller descriptions of special topics. Of course, this may not always be convenient. For example, Calot's method of applying plaster jackets is cited in such brief form and without illustrations that one would have to consult the original French article to obtain necessary details. Two pages, instead of a paragraph, devoted to this successful method would not be out of place. The increasing use of silk ligaments and Lange's preparation of the same are not given enough prominence. However, the book is a model of clearness and exact statements, with many illustrations, both original and otherwise, which are always to the point. There are no special chapters on medical gymnastics, on the use of Plaster of Paris, or on the *x*-ray as applied to orthopedics. In general, we can recommend the volume heartily as a standard textbook and as an attractive addition to any medical library.

---

A Fine Line of Sterilized Solutions.—Hermetically sealed glass ampoules containing sterilized solutions of important drugs for hypodermic use have assumed a commanding place in medicine in a comparatively short period of time. Two or three years ago, seeing the tendency in this direction, Parke, Davis & Co. brought out a modest line of something like a half-dozen formulas, notable among them being solutions of Adrenalin, Codrenin, and Cacodylate of Sodium. From this small beginning the line has expanded until now the company announces a total of about twenty distinct formulas. The full list, we understand, is now appearing in display advertisements in the leading medical journals of the country. Physicians who are interested in this advance in hypodermic medication—and every physician ought to be—will do well to search out these advertisements and familiarize themselves with the comprehensive line of solutions therein offered.

Solutions provided by the glaseptic ampoule, it is obvious, have several advantages over those prepared in the ordinary manner. They are ready for immediate use: there is no necessity to wait until water can be sterilized and cooled. Accuracy of dose is ensured, each ampoule containing a definite quantity of medicament. The solutions are aseptic; they are permanent.

